# Patient-Reported Outcomes After Surgical Treatment of Early Osteoarthritis of the First Carpometacarpal Joint

**DOI:** 10.1177/15589447221093669

**Published:** 2022-05-13

**Authors:** Merel H. J. Hazewinkel, Peter DiGiovanni, Satoshi Miyamura, Jonathan Lans, Neal C. Chen, Kiera Lunn, Jesse B. Jupiter

**Affiliations:** 1 Harvard Medical School, Boston, MA, USA

**Keywords:** arthritis, arthroplasty, carpometacarpal joint, patient reported outcomes, Wilson osteotomy

## Abstract

**Background:**

The goals of this study are to describe the reoperation rates in patients who underwent Wilson osteotomy compared with patients who underwent carpometacarpal (CMC) arthroplasty for early-stage arthritis and to evaluate the factors influencing the patient-reported outcomes.

**Methods:**

Retrospectively, 52 patients who underwent surgery for stage I/II osteoarthritis of the thumb carpometacarpal were identified, consisting of 17 (33%) patients who underwent Wilson osteotomy and 35 (67%) who underwent carpometacarpal arthroplasty. A total of 28 (55%) patients completed the outcome questionnaires, consisting of 11 (39%) patients who underwent Wilson osteotomy and 17 (61%) patients who underwent carpometacarpal arthroplasty. We performed a multivariable linear regression model to identify factors associated with the Numeric Rating Scale (NRS) pain intensity at final follow-up.

**Results:**

Among the patients who underwent CMC arthroplasty, 2 had a reoperation. Among the patients who underwent Wilson osteotomy, 3 had a reoperation. Among the patients who completed the outcome questionnaires, the median quick Disabilities of the Arm, Shoulder and Hand score was 10 and the median NRS Pain Intensity score was 0. In multivariable analysis, the postoperative Patient-Reported Outcomes Measurement Information System Pain Interference (PROMIS PI) was independently associated with higher postoperative NRS pain scores.

**Conclusion:**

In younger patients with stage I/II CMC osteoarthritis, Wilson osteotomy may be a reasonable alternative to CMC arthroplasty. Outcomes were similar between both groups at mid-term follow-up, with only a slightly higher pain score in the osteotomy group. In patients with stage I/II carpometacarpal osteoarthritis, the PROMIS PI is the main factor indicating successful outcomes.

## Introduction

Osteoarthritis (OA) of the carpometacarpal (CMC) joint of the thumb is highly prevalent, affecting 7% of men and 15% of women.^1,2^ Although most cases are not limiting, some patients choose to seek care for this condition. Conservative treatment options include splinting, occupational therapy, or injection with steroids.^3,4^

Trapeziectomy followed by ligament reconstruction and tendon interposition, also referred to as CMC arthroplasty, is a well-established procedure to treat CMC osteoarthritis in older patients.^5-7^ However, in symptomatic patients with early-stage CMC osteoarthritis, a Wilson osteotomy—a closing wedge osteotomy of the thumb metacarpal—is an option for treatment.^8,9^ Biomechanical studies suggest that excising a wedge of the metacarpal changes the angle of the saddle-back structure of the joint. This reduces the load through the dorsal trapeziometacarpal joint and increases the stability of the dorsal CMC-joint ligament.^10-12^ Published results are encouraging, but these are primarily series with limited comparisons.^8,13,14^ A study by Atroshi et al^15^ comparing metacarpal osteotomy with CMC arthroplasty in 17 patients indicates that first metacarpal osteotomy as a surgical option in trapeziometacarpal arthrosis should be limited to patients with early-stage CMC osteoarthritis.

The goals of this study are to report the reoperation rates in patients who underwent Wilson osteotomy compared with patients who underwent CMC arthroplasty for early-stage arthritis and to evaluate the factors influencing the patient-reported outcomes of these patients to improve future surgical decision-making. Factors that will be evaluated include patient demographics, comorbidities, and prior conservative treatment of the affected thumb.

## Materials and Methods

**Figure 1.**
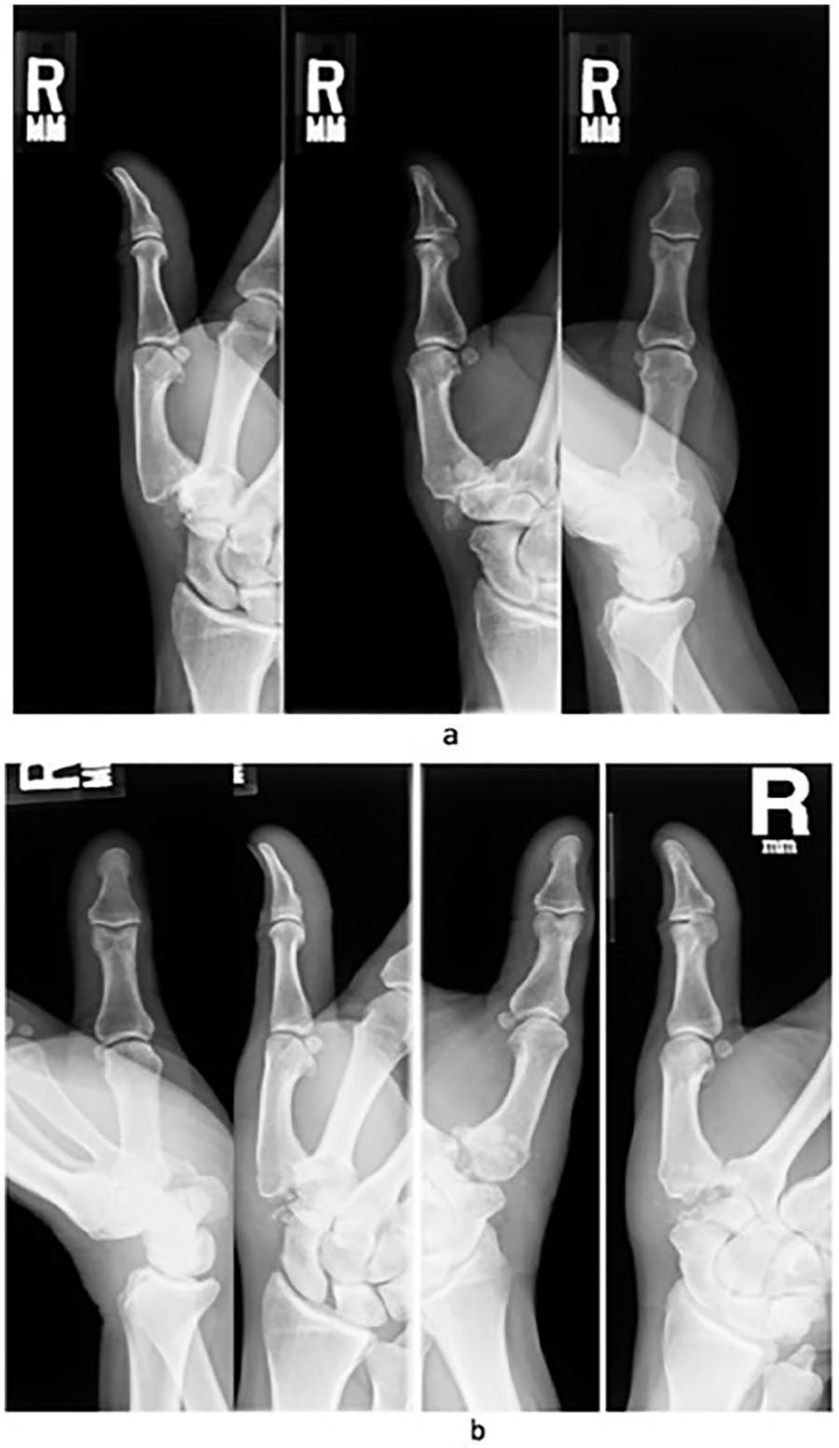
Radiographs of a patient with carpometacarpal arthroplasty: (a) preoperative and (b) postoperative.

**Figure 2.**
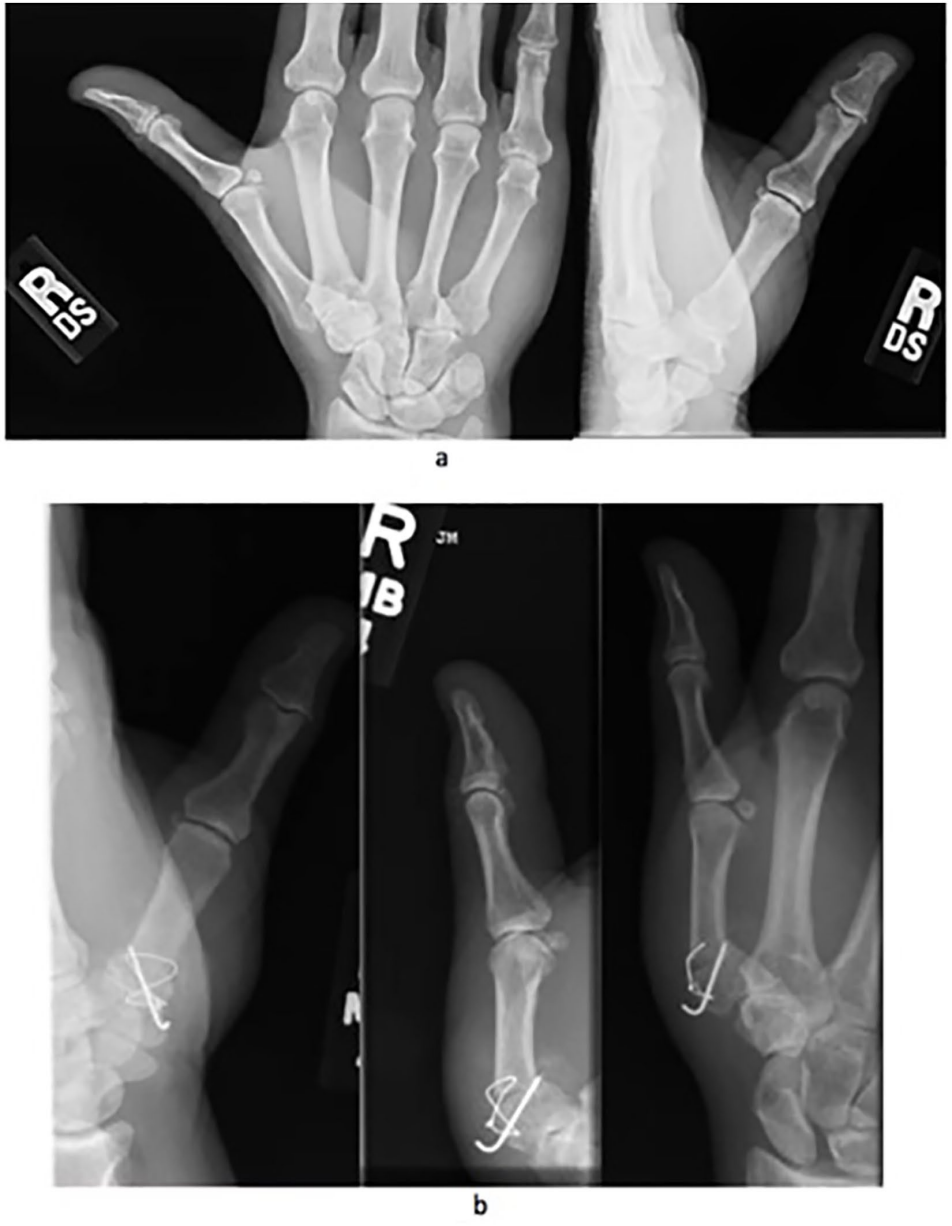
Radiographs of a patient with Wilson osteotomy: (a) preoperative and (b) postoperative.

**Figure 3.**
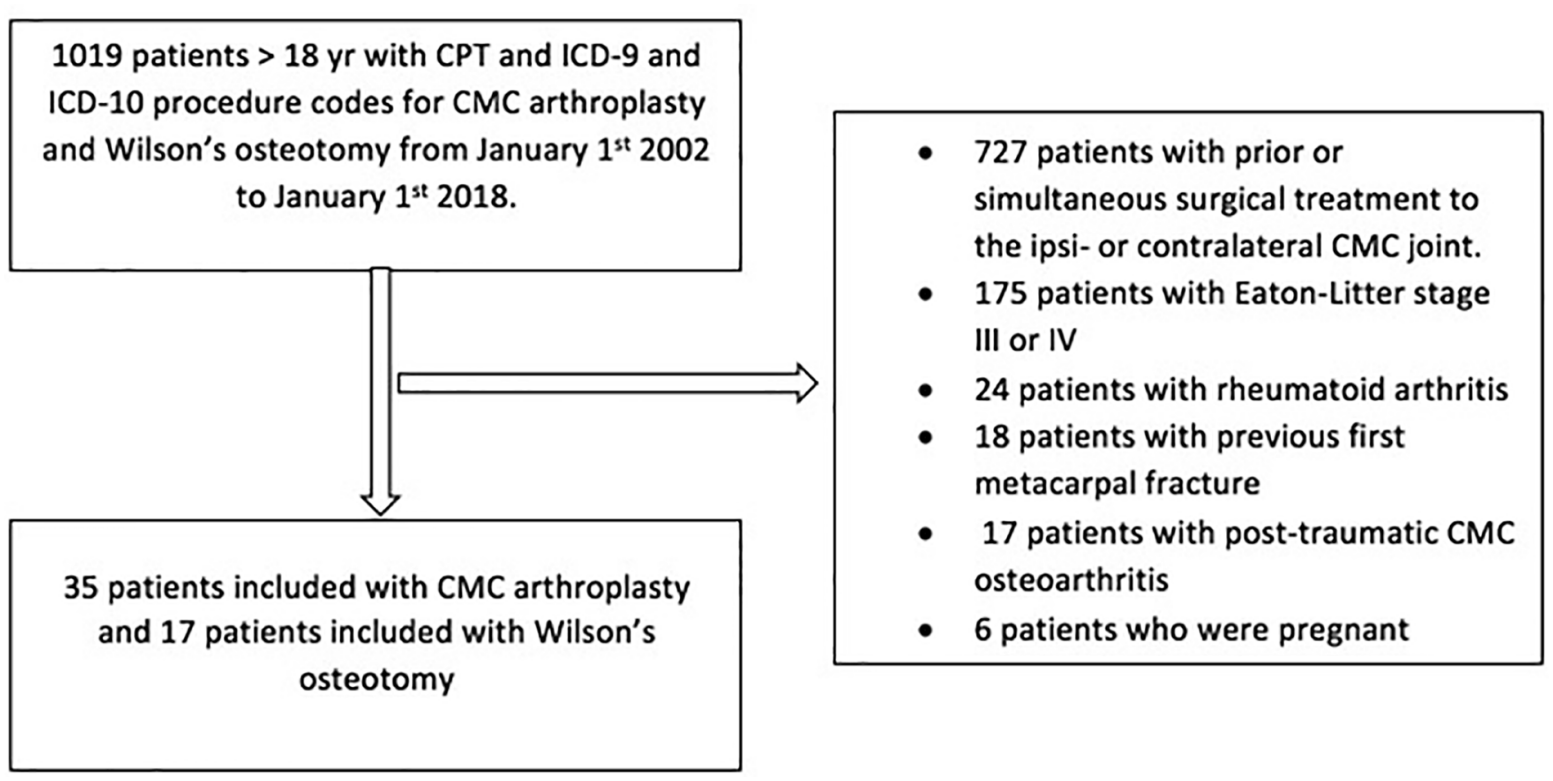
Flowchart of patient inclusion. *Note.* CPT = Current Procedural Terminology; ICD-9 = International Classification of Diseases, Ninth Revision; ICD-10 = International Classification of Diseases, Tenth Revision; CMC = carpometacarpal.

After institutional review board approval, we retrospectively identified patients who underwent either Wilson osteotomy or CMC arthroplasty using Current Procedural Terminology codes and International Classification of Diseases, Ninth and Tenth Revision (Supplemental Material 1). All relevant orthopedic encounters from January 1, 2002, to January 1, 2018, at a single institutional system in the Northeastern United States were verified by manual chart review (n = 1019). We included all adult patients who had a primary Wilson osteotomy or CMC arthroplasty for Eaton-Littler stage I/II CMC osteoarthritis (Figures 1 and 2). We excluded patients who underwent prior surgical treatment to the ipsilateral or contralateral CMC joint (n = 727), patients who were pregnant (n = 6), patients with a previous first metacarpal fracture (n = 18), patients with rheumatoid arthritis (n = 24), and patients with posttraumatic CMC osteoarthritis (n = 17). We identified 17 patients who underwent a Wilson osteotomy. All patients had Eaton-Littler stage I/II CMC osteoarthritis on radiographic review. Radiographs of all patients who underwent CMC arthroplasty (n = 210) were independently analyzed by a nontreating orthopedic hand surgeon to determine the CMC osteoarthritis stage. Thirty-five patients who underwent CMC arthroplasty for Eaton-Littler stage I/II CMC arthritis were identified. We only included the first CMC joint that was surgically treated in the analyses to avoid double-counting of potential factors when performing the analyses (Figure 3).

We performed a manual chart review to collect data including demographics (sex, age, race, occupation), comorbidities (smoking, diabetes, oral steroid use, carpal tunnel syndrome ipsilateral, trigger digits ipsilateral), and prior conservative treatment of the affected thumb (CMC injection, splint, pain medication) and affected hand (dominant vs nondominant). Preoperative radiographic explanatory variables including scaphotrapeziotrapezoidal osteoarthritis, metacarpal subluxation, and metacarpophalangeal (MCP) joint hyperextension >30° were evaluated by an orthopedic hand surgeon using conventional radiographs. These data were available in 48 (92%) of 52 patients. In addition, we collected data regarding postoperative symptoms (numbness), complications (infection, wound dehiscence, neuroma, and nonunion), and reoperations. Wound dehiscence was defined as partial or complete separation of previously approximated wound edges due to a failure of proper wound healing. Reoperation was defined as any unplanned additional thumb surgery following either Wilson osteotomy or CMC arthroplasty. The medical chart follow-up was the time from initial surgery to last clinical visit at one of the institutional hospitals, and the questionnaire follow-up was determined as the time from surgery to questionnaire completion.

### Long-Term Patient-Reported Outcomes

To evaluate long-term outcomes, we contacted all patients who were not deceased (n = 51 of 52) by mail to invite them to participate in our study to complete the questionnaires. The patients who did not fill in the questionnaires were contacted by phone by one of the researchers, and an option to complete the questionnaires via telephone was given. Eight (16%) patients declined participation, and 15 (29%) patients could not be contacted. A total of 28 (55%) patients completed the outcome questionnaires, consisting of 11 (39%) patients who underwent Wilson osteotomy and 17 (61%) patients who underwent CMC arthroplasty. The compared responders’ and nonresponders’ demographics were similar, except that there were more manual laborers in the nonresponse group (Supplemental Table S1). Study data were collected and managed using REDCap (Research Electronic Data Capture) electronic data capture tools hosted at our institution.^16^

### Patient-Reported Outcomes

Patients postoperatively completed the quick Disabilities of the Arm, Shoulder and Hand (QuickDASH) and the Patient-Reported Outcomes Measurement Information System Pain Interference (PROMIS PI) Computer Adaptive Test. The QuickDash consists of 11 items addressing the symptoms and functionality of patients with any or multiple disorders of the upper extremity.^17^ A QuickDASH score of 10 ± 15 is considered to be a normative value from the general population, with higher scores representing increased disability due to the underlying condition.^18^ The PROMIS PI was included as an explanatory variable as prior studies suggest pain interference affects patient-reported outcomes. It measures the extent to which pain interferes with social cognitive, emotional, physical, and recreational activities and was developed based on the item response theory.^19^ The PROMIS PI score has a general US population–based mean *T*-score of 50, with higher score representing increased impact of pain on daily activities.^20^ In addition, subjects were asked to report the Numeric Rating Scale (NRS) pain intensity in the past week along with a custom questionnaire assessing whether patients underwent reoperation.

### Statistical Analysis

Categorical data were presented as frequencies and percentages, and continuous data were expressed either as means and standard deviations or as median and interquartile range (IQR). To compare patients who underwent Wilson osteotomy with patients who underwent CMC arthroplasty, we used the Fisher exact test for categorical variables and the Student *t* test or Mann-Whitney *U* test depending on the normality of continuous variables. To evaluate the correlation between the outcomes (QuickDASH and NRS pain) and the patient characteristics, we used the Mann-Whitney *U* test for nonparametric continuous variables and the Spearman rank correlation coefficient for age and the PROMIS PI. We included all explanatory variables with *P* < .05 in a multivariable linear regression model to identify independent factors associated with the NRS pain intensity. Statistical significance was defined as *P* < .05.

## Results

### Study Population

We included 52 patients, namely, 17 (33%) patients who underwent Wilson osteotomy and 35 (67%) patients who underwent CMC arthroplasty. The mean age was 56 ± 8.2 years, and most patients were women (n = 34, 65%). The median chart follow-up was 5.5 years (IQR = 2.4-9.5). The median questionnaire follow-up was 6.4 (4.8) years. Sixteen (31%) patients had a bilateral thumb surgery (Table 1).

**Table 1.** Study Population.

Variable	All patients (n = 52)	Wilson (n = 17)	Arthroplasty (n = 35)	*P* value
	n (%)	n (%)	n (%)	
Age, mean (SD), y	56 (8.2)	52 (7.4)	58 (7.7)	**<.05** *
Male sex, No. (%)	18 (35)	8 (44)	10 (56)	.22**
Diabetes mellitus, No. (%)^a^	7 (14)	4 (57)	3 (43)	.20**
Smoking, No. (%)^b^	5 (10)	3 (60)	2 (40)	.33**
Manual labor, No. (%)^c^	6 (14)	3 (50)	3 (50)	.66**
Prior treatment				
Prior splint, No. (%)^b^	49 (67)	9 (27)	24 (73)	.20**
Prior CMC injection, No. (%)^b^	26 (53)	6 (23)	20 (77)	.08**
Prior pain medication, No. (%)^d^	21 (42)	5 (24)	16 (76)	.24**
Preoperative radiographic explanatory variables				
STT osteoarthritis, No. (%)^e^	1 (1.9)	0	1 (100)	.71**
Metacarpal subluxation, No. (%)^e^	12 (23)	3 (25)	9 (75)	.51**
MCP hyperextension, No. (%)^f^		2 (100)	0	.08**
Surgery dominant hand, No. (%)^d^	28 (56)	12 (43)	16 (57)	.23**
Procedure, No. (%)				—
CMC arthroplasty	35 (67)	—	—	
Wilson osteotomy	17 (33)	—	—	
Bilateral thumb surgery, No. (%)	16 (31)	3 (19)	13 (81)	.21**
Follow-up time, median (IQR), y	5.5 (2.4-9.5)	5.4 (2.4-9.4)	5.5 (2.4-9.7)	.48*

*Note.* CMC = carpometacarpal; IQR = interquartile range; STT = scaphotrapeziotrapezoidal; MCP = metacarpophalangeal. *Missing values*:

a =1 missing.

b =3 missing.

c =6 missing.

d =2 missing.

e =4 missing.

f =5 missing.

* Using Student *t* test.

** Using Fisher exact test.

Bold represents *P* < .05.

The mean age was higher among patients who underwent CMC arthroplasty compared with those who underwent Wilson osteotomy (52 ± 7.4 vs 58 ± 7.7, *P* < .001) (Table 1). Long-term postoperative numbness was more common after Wilson osteotomy (n = 4 [11%] vs n = 7 [41%], *P* < 0.05) compared with CMC arthroplasty (Table 2). Among the patients who underwent Wilson osteotomy, 3 underwent reoperation: 2 underwent implant removal for symptomatic implants and 1 with Eaton-Litter stage II arthritis underwent trapezium resection arthroplasty for pain. Among the patients who underwent CMC arthroplasty, 2 had a reoperation: 1 underwent a revision arthroplasty for pain and 1 underwent arthrodesis of the MCP joint for hyperextension after arthroplasty. In our cohort, 3 patients had Eaton-Littler stage I CMC osteoarthritis, and all underwent CMC arthroplasty. The remaining 49 patients had Eaton-Litter stage II.

**Table 2.** Postoperative Outcomes of Wilson Osteotomy Versus Carpometacarpal Arthroplasty.

Variable	Wilson (n = 17)	Arthroplasty (n = 35)	*P* value
	No. (%)	No. (%)	
Infection, No. (%)^a^	1(5.9)	0	.33*
Wound dehiscence, No. (%)^a^	0	0	>.99*
Numbness, No. (%)^a^	7(41)	4(11)	**<.05** *
Neuroma, No. (%)^a^	0	0	>.99*
Nonunion, No. (%)^a^	0	0	>.99*
Reoperation, No. (%)	3(18)^c^	2(5.7)	.32*

*Note. Missing values*:

a =1 missing.

* Using Fisher exact test.

** One patient underwent conversion to trapezial resection arthroplasty.

Bold represents *P* < .05.

Twenty-eight patients completed the questionnaires at a median of 8.3 (IQR = 4.8-13) years following index surgery. There was no difference in the follow-up period between the patients who underwent Wilson osteotomy and the patients who underwent CMC arthroplasty. Two (22%) patients had a reoperation for pain, consisting of 1 patient who underwent CMC arthroplasty and 1 patient who underwent Wilson osteotomy. The median QuickDASH score was 10 (IQR = 4.5-27) and the median NRS pain intensity score was 0 (IQR = 0-1) for all the patients who were able to obtain follow-up. The median NRS pain score among patients who underwent CMC arthroplasty was 0 (IQR = 0-0), and the median NRS pain score among patients who underwent Wilson osteotomy was 1 (IQR = 0-5) (*P* < .05).

In bivariate analysis, higher QuickDASH scores were associated with higher PROMIS PI scores (*R* = 0.82, *P* < .05) (Table 3). In multivariable analysis, the PROMIS PI was independently associated with higher NRS pain scores (β = 0.13, *P* < .05) (Table 4).

**Table 3.** Patient-Reported Outcomes.

Variable	QuickDASH		NRS pain intensity	
	Median (IQR)	*P* value	Median (IQR)	*P* value
All patients (n = 28)	10 (4.5-27)		0 (0-1)	
PROMIS pain interference	0.82	**<.05** *	0.75	**<.05** *
Age	–0.22	.25*	–0.34	.08*
Sex		.80**		.55**
Male	10 (2.3-30)		0 (0-3)	
Female	10 (4.5-25)		0 (0-1)	
Diabetes mellitus^a^		.92**		.71**
Yes	9.1 (2.3-66)		0 (0-6)	
No	11 (4.5-27)		0 (0-1)	
Race		.30**		.46**
Caucasian	9.1 (4.5-25)		0 (0-1)	
Hispanic	43 (43-43)		3 (3-3)	
Smoking^b^		.61**		.42**
Yes	22 (4.5-50)		0.5 (0-3.5)	
No	10 (4.4-20)		0 (0-1)	
Manual labor^c^		.077**		.23**
Yes	70 (70-70)		5 (5-5)	
No	9.1 (4.5-16)		0 (0-1)	
Prior carpal tunnel syndrome ipsilateral		.63**		.96**
Yes	6.8 (2.3-38)		0 (0-3)	
No	11 (4.5-27)		0 (0-1)	
Prior trigger finger ipsilateral		.70**		>.99**
Yes	9.1 (0-30)		0 (0-3)	
No	11 (4.5-66)		0 (0-1)	
Prior splint^b^		.064**		.16**
Yes	8.0 (2.3-16)		0 (0-0)	
No	14 (11-34)		0 (1-3)	
Prior CMC injection^b^		.43**		.32**
Yes	16 (4.5-43)		0 (0-3)	
No	9.1 (4.5-14)		0 (0-1)	
Prior pain medication^d^		.34**		.98**
Yes	14 (9.1-34)		0 (0-1)	
No	9.1 (4.5-25)		0 (0-1)	
Procedure		.19**		**<.05** *
Arthroplasty	9.1 (4.5-16)		0 (0-0)	
Wilson	14 (6.8-50)		1 (0-5)	
Surgery dominant hand^d^		.56**		.97**
Yes	13 (6.8-30)		0 (0-1)	
No	9.1 (2.3-25)		0 (0-3)	
Bilateral thumb surgery		.31**		.81**
Yes	16 (4.5-30)		0 (0-3)	
No	9.1 (4.5-14)		0 (0-1)	

*Note.* DASH = Disabilities of the Arm, Shoulder and Hand; NRS = Numeric Rating Scale; IQR = interquartile range; PROMIS = Patient-Reported Outcomes Measurement Information System; CMC = carpometacarpal. *Missing values*:

a =1 missing.

b =3 missing.

c =6 missing.

d =2 missing.

* Using Spearman rank correlation coefficient.

** Using Mann-Whitney *U* test.

Bold represents *P* < .05.

**Table 4.** Multivariable Linear Regression.

Factors associated with NRS pain intensity				
Variable	Coeff.	SE	95% CI	*P* value
PROMIS pain interference	0.13	0.026	0.083 to 0.19	**<.05**
Procedure	–0.61	0.52	–1.7 to 0.46	.52

*Note.* NRS = Numeric Rating Scale; CI = confidence interval; PROMIS = Patient-Reported Outcomes Measurement Information System.

All explanatory variables with *P* < .05 were included in the multivariable analysis. Bold represents *P* < .05.

## Discussion

This study evaluated the patient-reported outcomes following Wilson osteotomy and CMC arthroplasty in patients with early osteoarthritis of the thumb. Two of the patients who underwent CMC arthroplasty had a reoperation. Among the patients who underwent Wilson osteotomy, 3 had a reoperation, of which only 1 consisted of conversion to CMC arthroplasty. Long-term postoperative numbness was more common after Wilson osteotomy, possibly because the branches of the radial sensory nerve are at risk. The 28 patients who completed the questionnaire at a median of 8.3 years reported a median QuickDASH score of 10 and a median NRS pain intensity score of 0. In multivariable analysis, higher PROMIS PI scores were independently associated with higher NRS pain scores.

The results of our study should be interpreted in light of its limitations. First, a manual chart review was performed to collect data; therefore, our analysis depends on the accuracy of the data reported in the chart. Second, only 55% of patients completed the questionnaires. However, this response rate is in line with other long-term follow-up hand surgery studies using similar methodology.^21-23^ In addition, there may be selection bias as we observed more nonmanual laborers in the responding cohort. We defined the follow-up time as the time from surgery to last clinical visit recorded in medical charts considering that this would minimize the loss of follow-up from patients who switched hospitals within our institutional system. However, it is a possibility that we underestimated the reoperation rate if patients underwent reoperation outside our institution. Also, the MCP joint hyperextension was evaluated solely using radiography, and the clinical hyperextension was not measured. Another limitation is that we did not obtain pinch or grip strength, which could be helpful in understanding the function that is not well captured in patient-rated outcome measures. Also, the PROMIS PI and NRS pain intensity score were only obtained postoperatively. Finally, we did not have adequate power to comment on equivalence between Wilson osteotomy and CMC arthroplasty.

In this study, higher NRS pain intensity scores were associated with Wilson osteotomy (0, IQR = 0-0 vs 1.0, IQR = 0-5, *P* < .05). Our results are in line with prior studies that report a median pain score of 1 (IQR: 1-6) and a median QuickDASH score of 9.1 (IQR: 0-79.6) after Wilson osteotomy.^8^ Similarly, prior studies report mean QuickDASH scores of 11.8 ± 14.3 and a mean pain score of 1.1-1.4 following CMC arthroplasty for Eaton-Litter stages II and III.^5,6^ The difference between the NRS pain scores may be that patients who underwent Wilson osteotomy were younger than those undergoing CMC arthroplasty and may place greater loads on the thumbs. It is important to note that this surgical scenario in a younger patient with mild arthritis is rare. Patients with mild CMC arthritis rarely elect surgery, and it is also uncommon for younger patients to elect arthroplasty.^5,6,8^ In addition, it is not clear whether the NRS pain score was similar at the median 8-year follow-up to the initial follow-up period or whether we are observing progression of arthrosis over time after Wilson osteotomy. If these results reflect progression of arthrosis, it is encouraging that the median NRS pain score is 1.

In the patients undergoing Wilson osteotomy and CMC arthroplasty for early-stage CMC osteoarthritis, higher PROMIS PI scores were associated with higher QuickDASH scores and higher NRS pain scores. The relationship between functional outcomes and PROMIS PI has been frequently described in upper extremity literature.^24,25^ Döring et al^26^ studied 84 new and follow-up patients who presented to an orthopedic outpatient clinic and found that the PROMIS PI was the strongest independent predictor of upper extremity disability measured by QuickDASH (β = 2.0, *P* < .05). In addition, there is strong evidence that the PROMIS PI also correlates well to coping in response to nociception.^27^ Pain intensity comes about from less effective coping strategies, and patients who report higher degrees of pain are more commonly offered surgery.^28-30^ Patients who elect surgery for lower radiographic stages of CMC arthritis may have greater pain interference than the general population, and our data suggest that this factor is more indicative of outcome relative to the choice of surgical treatment.

Among the patients who underwent Wilson osteotomy, 3 underwent reoperation, including 1 major reoperation. Bachoura et al studied 32 thumbs in 28 patients with stage I, II, and III osteoarthritis who underwent Wilson osteotomy and reported that 7 patients (22%) underwent a reoperation. Reoperations included 6 CMC arthroplasties and 1 CMC arthroscopy.^31^ Among the patients who underwent CMC arthroplasty in the current study, 2 underwent reoperation, both being major reoperations. Cooney et al retrospectively evaluated more than 600 patients with Eaton-Litter stage II, III, and IV osteoarthritis who underwent CMC arthroplasty. They reported that 15 patients (2.5%) underwent reoperation following CMC arthroplasty.^32^ Although the reoperation rates following both Wilson osteotomy and CMC arthroplasty in early-stage CMC arthritis seemed similar, they appear to be higher compared with higher stages and are consistent with the concepts of Pain Interference.

We found that outcomes were similar between patients who underwent Wilson osteotomy and CMC arthroplasty at mid-term follow-up. For surgeons who are conflicted about performing CMC arthroplasty in a younger patient with mild arthritis that is refractory to nonoperative management, Wilson osteotomy may be a reasonable alternative. If outcomes are similar, in patients where preservation of pinch strength or durability is a concern, Wilson osteotomy may be advantageous compared with CMC arthroplasty; however, this theoretical advantage should be balanced against the risk of increased sensory nerve injury and reoperation for hardware removal.

In younger patients with stage I/II CMC osteoarthritis, Wilson osteotomy may be a reasonable alternative to CMC arthroplasty. Conversion to CMC arthroplasty after osteotomy was rare in our cohort, and outcomes were similar between both groups at mid-term follow-up with only a slightly higher pain score in the osteotomy group. It is also important to indicate patients carefully, as PROMIS PI was the primary factor associated with worse overall outcomes.”

## Supplemental Material

sj-docx-1-han-10.1177_15589447221093669 – Supplemental material for Patient-Reported Outcomes After Surgical Treatment of Early Osteoarthritis of the First Carpometacarpal Joint Click here for additional data file. Supplemental material, sj-docx-1-han-10.1177_15589447221093669 for Patient-Reported Outcomes After Surgical Treatment of Early Osteoarthritis of the First Carpometacarpal Joint by Merel H. J. Hazewinkel, Peter DiGiovanni, Satoshi Miyamura, Jonathan Lans, Neal C. Chen, Kiera Lunn and Jesse B. Jupiter in HAND

sj-docx-2-han-10.1177_15589447221093669 – Supplemental material for Patient-Reported Outcomes After Surgical Treatment of Early Osteoarthritis of the First Carpometacarpal Joint Click here for additional data file. Supplemental material, sj-docx-2-han-10.1177_15589447221093669 for Patient-Reported Outcomes After Surgical Treatment of Early Osteoarthritis of the First Carpometacarpal Joint by Merel H. J. Hazewinkel, Peter DiGiovanni, Satoshi Miyamura, Jonathan Lans, Neal C. Chen, Kiera Lunn and Jesse B. Jupiter in HAND
