# Supplementary material for: Patient-Reported Outcomes After Surgical Treatment of Early Osteoarthritis of the First Carpometacarpal Joint
Source: Hand (N Y). 2022 May 13;18(8):1275–83. doi: 10.1177/15589447221093669 (PMC10617478; doi:10.1177/15589447221093669)
Supplement: sj-docx-1-han-10.1177_15589447221093669 – Supplemental material for Patient-Reported Outcomes After Surgical Treatment of Early Osteoarthritis of the First Carpometacarpal Joint [file sj-docx-1-han-10.1177_15589447221093669.docx]

**CPT codes**

25447: Arthroplasty, interposition, intercarpal or carpometacarpal joints

25210: Carpectomy; 1 bone

26480: Transfer or transplant of tendon, carpometacarpal area or dorsum of hand; without free graft, each tendon

26483: Transfer or transplant of tendon, carpometacarpal area or dorsum of hand; with free tendon graft (includes obtaining graft), each tendon

26565: Osteotomy; metacarpal, each

**ICD-9 procedure codes**77.24: Wedge osteotomy of carpals and metacarpals

77.34: Other division of carpals and metacarpals

77.84: Other partial ostectomy of carpals and metacarpals
