# Supplementary material for: Patient-Reported Outcomes After Surgical Treatment of Early Osteoarthritis of the First Carpometacarpal Joint
Source: Hand (N Y). 2022 May 13;18(8):1275–83. doi: 10.1177/15589447221093669 (PMC10617478; doi:10.1177/15589447221093669)
Supplement: sj-docx-2-han-10.1177_15589447221093669 – Supplemental material for Patient-Reported Outcomes After Surgical Treatment of Early Osteoarthritis of the First Carpometacarpal Joint [file sj-docx-2-han-10.1177_15589447221093669.docx]

|  |  |  |  |
| --- | --- | --- | --- |
| Supplemental Table S1: Responders vs Non-responders | | | |
|  |  |  |  |
|  | **Non-responders (n=23)** | **Responders (n=28)** |  |
| **Variable** | **n(%)** | **n(%)** | **P value** |
| **Age, mean (SD), years** | 57(9.1) | 56(7.5) | 0.87* |
| **Male sex, n(%)** | 8(35) | 10(36) | >0.99 |
| **Diabetes Mellitus, n(%) ^a^** | 4(17) | 3(11) | 0.69** |
| **Oral steroid use, n(%) ^b^** | 0 | 3(12) | 0.24** |
| **Race, n(%) ^c^** |  |  | >0.99** |
| Caucasian | 23(100) | 25(96) |  |
| Hispanic | 0 | 1(3.9) |  |
| **Smoking, n(%) ^c^** | 1(5.0) | 4(14) | 0.39** |
| **Manual labor, n(%) ^d^** | 5(31) | 1(3.9) | **<0.05**** |
| **Procedure, n(%)** |  |  | 0.38** |
| CMC arthroplasty | 17(74) | 17(61) |  |
| Wilson's osteotomy | 6(26) | 11(39) |  |
| **Surgery dominant hand, n(%) ^b^** | 14(64) | 14(50) | 0.40** |
| Missing values | * using Student's t-test |  |  |
| ^a^=1 missing | **using Fisher's exact test | |  |
| ^b^=2 missing | Bold is p<0.05 |  |  |
| ^c^=3 missing |  |  |  |
| ^d^=6 missing |  |  |  |
